# Initial Audit Investigating Physical Health Screening and Management of Neuropsychiatric Manifestations in Parkinson’s Disease (PD), Parkinson’s Disease Dementia (PDD) and Dementia With Lewy Bodies (DLB) on the Mental Health Services for Older Persons (MHSOP) Wards

**DOI:** 10.1192/bjo.2025.10658

**Published:** 2025-06-20

**Authors:** Mohammed Qureshi, Taofeeq Elias, Olaomopo Ikuforiji, Chidinma Okafor, Samuel Cohen

**Affiliations:** 1 Leicestershire Partnership NHS Trust, Leicester, United Kingdom; 2 University Hospitals of Leicester NHS Trust, Leicester, United Kingdom

## Abstract

**Aims:** To improve the overall quality of healthcare for mental health inpatients with a diagnosis of PD, PDD or DLB (with neuropsychiatric manifestations such as depression, agitation, psychosis or cognitive deficits), by improving: (a) physical healthcare screening and (b) the collaborative approach between inpatient mental health teams and PD specialists.

**Methods:** Our audit included 24 patients admitted to five MHSOP wards in Leicestershire, between 13/12/2020 and 27/10/2022. A list was obtained from the e-prescribing team, of patients on specific psychotropic or parkinsonian medications that were identified in advance by the audit team. An online audit tool was created which consisted of 17 questions relating to patient care. Three resident doctors conducted the retrospective data collection after being briefed by the audit lead, to ensure consistency in the data collection process.

**Results:** Antipsychotics were considered in 11/11 (100%) patients who had psychotic symptoms. 20/24 (83%) patients experiencing neuropsychiatric issues had bloods taken to rule out acute physical health causes. In 21/24 (88%) cases, an electrocardiogram was conducted prior to commencing an antipsychotic or cholinesterase inhibitor. Cholinesterase inhibitors were considered in 13/17 (76%) of patients with dementia.

Optimising dopaminergic therapy for those with PD was poor, with 8/18 (44%) patients having an intervention involving dopaminergic medications. For 5/24 (21%) patients, advice was sought from PD specialists (2 with geriatricians, 2 with neurologists and 1 with a PD specialist nurse). Of these, 3 (13%) were assessed in person by the PD specialist. In 8/24 (33%) cases, follow-up with a PD specialist was considered by the ward. There was poor compliance in screening for autonomic dysfunction in the context of PD (e.g. constipation, urinary dysfunction and orthostatic hypotension).

**Conclusion:** As stated in the DIAMOND-Lewy toolkit, “many patients with PDD or DLB do not receive the best possible management”. Furthermore, the pathway for obtaining PD specialist input is unclear, although the collaboration between inpatient mental health teams and PD specialists is potentially vital for holistic care.

This audit demonstrates areas for improvement in terms of physical health screening (particularly the domains of autonomic dysfunction) and in the collaboration with PD specialists, with view to providing comprehensive healthcare for mental health inpatients with PD, PDD or DLB. Interventions prior to re-auditing will include raising awareness amongst inpatient teams of the need to review parkinsonian medications and of screening for autonomic dysfunction, as well as discussions with PD specialists regarding how collaboration can be improved and streamlined.

